# Associations between sugar-sweetened beverage consumption, weight-adjusted-waist index, with psychological symptoms: a cross-sectional survey of adolescents in mainland China

**DOI:** 10.3389/fpsyt.2025.1558919

**Published:** 2025-03-28

**Authors:** Rui Wang, Shihua Guo, Guangyan Yang, Jun Li

**Affiliations:** ^1^ School of Sports and Health Management, Henan Finance University, Zhengzhou, China; ^2^ Zhengzhou University of Science and Technology Sports College, Zhengzhou, China; ^3^ School of Physical Education and Sports, Chizhou University, Chizhou, China

**Keywords:** sugar-sweetened beverage consumption, weight-adjusted-waist index, psychological symptoms, associations, China

## Abstract

**Background:**

The prevalence of psychological symptoms (emotional problems, behavioral problems, social adjustment difficulties) in adolescents continues to increase and has become a major concern in various countries. However, few studies have been conducted on the association between sugar-sweetened beverage (SSB) consumption, weight-adjusted waist index (WWI), and psychological symptoms in adolescents. The present study provides a reference for the prevention and intervention of psychological symptoms in Chinese adolescents.

**Methods:**

A multistage stratified whole cluster random sampling method was used to assess psychological symptoms, SSB consumption, weight, waist circumference, and demographic information in 44,675 adolescents aged 12-17 years in mainland China in 2023. The associations between SSB consumption, WWI, and psychological symptoms among adolescents were analyzed using logistic regression model analysis and ordered logistic regression analysis with the generalized linear model.

**Results:**

The prevalence of psychological symptoms among Chinese adolescents was 20.9%, with boys (21.4%) having a higher prevalence than girls (20.5%), and the difference was statistically significant (*χ* ^2^ value of 5.687, *P* < 0.05). The proportion of adolescents with SSB consumption >4 times/week was 14.5%, and the WWI value was 9.36 ± 1.13. Ordered logistic regression analysis showed that, using SSB consumption <1 times/week and WWI quartile of Q1 as the reference group, adolescents with SSB consumption >4 times/week and WWI quartile of Q4 had the highest risk of developing psychological symptoms (OR=2.01, 95% CI:1.76-2.30) (*P <*0.001). The same trend was observed in boys (OR=1.67, 95% CI: 1.39-2.00) and girls (OR=2.68, 95% CI: 2.17-3.31) (*P <*0.001).

**Conclusions:**

The prevalence of adolescent psychological symptoms is high in mainland China and is associated with SSB consumption and WWI. Effectively reducing SSB consumption and WWI plays a positive role in the prevention and intervention of adolescent psychological symptoms.

## 1 Introduction

Weight-adjusted-waist index (WWI) is a new type of obesity index, which is effective in assessing human obesity (1). The WWI is calculated by dividing waist circumference (cm) by the square root of weight (kg), thus normalizing waist circumference to weight (2). This index combines the benefits of waist circumference while attenuating the correlation with body mass index (BMI), making it possible to assess the body’s fat mass and muscle mass at the same time (3). Weight-adjusted-waist index was found to have higher sensitivity for assessing the occurrence of chronic diseases compared to other indicators of body fatness (4). The WWI was proposed to more accurately assess the relationship between obesity and cardiovascular disease (CVD) risk (5). While traditional obesity metrics such as BMI and waist circumference are also correlated with CVD risk, they do not clearly distinguish between muscle mass and fat mass (6). Elevated WWI reflects a state of excessive body fat accumulation and increased muscle mass loss, which is associated with the development of cardiovascular disease (7). A limited number of studies have found a strong association between WWI and adult mental health as well, while few studies have found a link between WWI and adolescent mental health (8, 9).

Sugar-sweetened beverage (SSB) overconsumption has become a major health threat to adolescents and is of worldwide concern (10). A survey of adolescent SSB consumption in 185 countries worldwide showed that adolescents in 56 (30.3%) countries had an average SSB consumption of ≥7 servings per week, representing 238 million adolescents, or 10.4% of the global young population (11). Studies found significant associations between SSB consumption and obesity, osteoporosis, dental health, executive functioning, and chronic disease in adolescents and unanimously called for SSB consumption to be reduced in adolescents to promote healthy development (12–15). A study has shown that the prevalence of overweight and obesity is 14% higher among in-school adolescents who drink sugary beverages more than once a day compared to those who don’t drink them at all (16). An analysis of surveys in different countries found significant differences in obesity rates and SSB consumption among adolescents in different countries and regions, with the prevalence of obesity ranging from 3.3% in Cambodia to 64.0% in Niue, and the proportion of adolescents who consumed SSB one or more times per day in school ranging from 3.3% in Iceland to 79.6% in Niue (17). Sugar-sweetened beverage consumption varies between countries and poses a serious threat to adolescent health. However, past studies have focused on the relationship between SSB consumption and physical illnesses, and limited research has been conducted on the relationship between SSB consumption and mental health (18). Limited studies have found that excessive SSB consumption in adolescents was associated with an increased prevalence of depression and anxiety (19, 20). Past studies have focused on groups of adolescents in developed countries and have investigated limited geographic areas and samples that are underrepresented, while fewer studies have been conducted on adolescents in developing countries.

The psychological symptoms of adolescents in this study were mainly emotional problems, behavioral problems and social adjustment difficulties. The prevalence of psychological symptoms is spreading rapidly across the globe, especially in developing countries, and poses a serious threat to schooling and healthy development during adolescence (21). Data show that globally, the average prevalence of mental disorders in individuals aged 5-24 years is 11.63%, the overall prevalence of mental disorders in the age group of 10-14 years is 13.96%, and in the age group of 15-19 years is 13.63%, and the prevalence of mental disorders in adolescents and young people in the age group of 10-19 years is comparatively high, which should be given sufficient attention and concern (22). It has also been shown that the effects of psychological symptoms in adolescence carry over into adulthood and pose a serious threat to adult health (23). Several past studies have found that the prevalence of psychological symptoms in adolescents is influenced by a combination of factors, such as sleep quality, obesity status, family factors, duration of exercise, and academic stress (24–26). However, it is noteworthy that past studies on obesity and adolescent psychological symptoms have mainly focused on conventional indicators such as BMI and waist circumference, while fewer studies have addressed the association between WWI and psychological symptoms (27). Based on past studies, it can be found that there is an association between adolescent SSB consumption and WWI and psychological symptoms. However, unfortunately there are no studies that have found the relationship between the combined effects of SSB consumption and WWI and psychological symptoms in Chinese adolescents.

China is a vast country, spanning from east to west and from north to south, and the prevalence of psychological symptoms among adolescents varies greatly from region to region (28). In this study, SSB consumption, WWI, and psychological symptoms were assessed in adolescents from different regions of mainland China. The aim was to analyze the correlations that exist between them and to provide references and lessons for the prevention and intervention of adolescents’ psychological symptoms in China.

## 2 Methods

### 2.1 Participants

**Figure 1:**
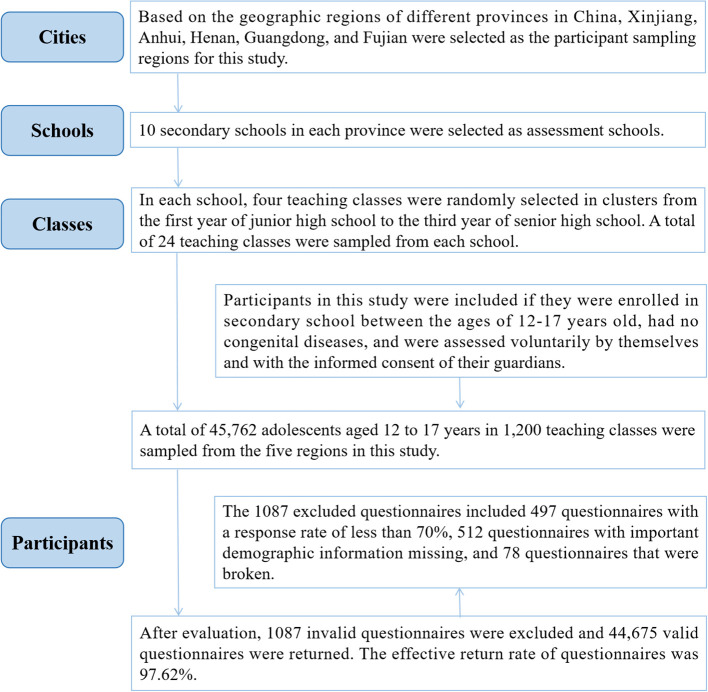
Participant extraction process.

In this study, 44,675 adolescents aged 12-17 years old in mainland China were assessed for psychological symptoms, SSB consumption, weight, waist circumference, and demographic information using a multistage stratified whole cluster random sampling method. The participant sampling process was as follows: First, based on the geographic regions of different provinces in China, Xinjiang, Anhui, Henan, Guangdong, and Fujian were selected as the participant sampling regions for this study. Second, 10 secondary schools in each province were selected as assessment schools. In each school, four teaching classes were randomly selected in clusters from the first year of junior high school to the third year of senior high school. A total of 24 teaching classes were sampled from each school. Participants in this study were included if they were enrolled in secondary school between the ages of 12-17 years old, had no congenital diseases, and were assessed voluntarily by themselves and with the informed consent of their guardians. A total of 45,762 adolescents aged 12 to 17 years in 1,200 teaching classes were sampled from the five regions in this study. After evaluation, 1087 invalid questionnaires were excluded and 44,675 valid questionnaires were returned. The 1087 excluded questionnaires included 497 questionnaires with a response rate of less than 70%, 512 questionnaires with important demographic information missing, and 78 questionnaires that were broken. The effective return rate of questionnaires was 97.62%. The participant sampling process is shown in **Figure 1**.

This study was conducted in accordance with the principles of the Declaration of Helsinki. Informed consent was obtained from parents or guardians before the assessment of participants in this study, and participants volunteered to be assessed for this study. This study was approved by the Ethics Committee of Chizhou University (202345612).

### 2.2 Weight-adjusted-waist index

WWI was calculated from the participants’ weight and waist circumference using the formula of waist circumference (cm) divided by the square root of weight (kg). Weight and waist circumference were assessed according to the methods required by the China National Survey on Students’ Constitution and Health (CNSSCH) (29). Waist circumference assessment results are accurate to 0.1 centimeters. Weight assessment results are accurate to 0.1 kg. Before the weight assessment, students were asked to wear as light clothes as possible for the test to minimize the assessment error. After the WWI in this study was stratified according to different ages and sex, their WWI was categorized into four categories according to quartiles, which were Q1 (<8.82), Q2 (8.82 ~ 9.38), Q3 (9.39 ~ 9.93), and Q4 (>9.93), in that order.

### 2.3 Sugar-sweetened beverage consumption

The assessment of SSB consumption in this study was based on the requirements of the questionnaire in the China National Survey on Students’ Constitution and Health (CNSSCH) (29). This program was initiated by the Chinese government to assess the health checkups and physical fitness of adolescents aged 6-22 years old nationwide every five years, to understand the health changes of adolescents nationwide in China. The present study focused on assessing participants’ SSB consumption over the past 30 days. The specific question was “In the past 30 days, how many times did you have sugar-sweetened beverages, such as all kinds of carbonated drinks, tea drinks, sugary juice drinks, coffee drinks, sugary nut drinks, sports drinks, lactic acid bacteria drinks, etc.?” In this study, SSB consumption was categorized as <1 times/week, 2-4 times/week, and >4 times/week (29). The questionnaire has been used in several studies in China, and it has good reliability and validity (30).

### 2.4 Psychological symptoms

Psychological symptoms of Adolescents in this study were assessed using the Multidimensional Sub-health Questionnaire of Adolescents(MSQA) (31). This questionnaire has been widely used among Chinese adolescents and has good reliability and validity for assessing adolescent psychological symptoms (32). The Cronbach’s alpha coefficient of this questionnaire is 0.86 (33). The questionnaire consists of 39 items, there are six options for each item, respectively, namely, “lasted >3 months”, “lasted >2 months”, “lasted >1 month”, “lasts >2 weeks”, ‘lasts >1 week’, ‘none or lasts ≤1 week’, each entry was a single choice. Participants were recorded with a score of 1 if they chose one of the first three entries and 0 if they chose one of the last questionnaire was divided into three dimensions: emotional problems, behavioral problems, and social adjustment difficulties. The total score of the three dimensions was the psychological symptoms score. The presence of psychological symptoms was assessed when 39 patients had a cumulative psychological symptom score of ≥8. Meanwhile, 18 of the 39 entries belonged to the dimension of emotional problems, emotional problems were defined if the score was ≥3. Eight items are part of the Behavioral Problems dimension, and a score of ≥1 defines the presence of a behavioral problem. The Social Adjustment Difficulty dimension has 13 entries and is defined as social adjustment difficulty if the score is ≥4.

### 2.5 Covariates

Covariates in this study included the father’s education level, mother’s education level, family economic level, sleep duration, and moderate-to-vigorous physical activity (MVPA). Father’s education level and mother’s education level are divided into four categories, elementary school, middle school, high school, university, and above. The family economic level is divided into four levels. They are < 2000 RMB/month, 2000-5000 RMB/month, 5001-8000 RMB/month, and > 8000 RMB/month. Sleep duration is calculated based on the participants’ sleep time and wake-up time, which is divided into three levels according to the classification of relevant studies, namely < 6 hours/day, 6-8 hours/day, and ≥ 8 hours/day (34). MVPA was derived from the entries in the CNSSCH questionnaire, which assessed participants’ average daily MVPA hours over the past 7 days, including 5 days on weekdays and 2 days on weekends, and categorized their MVPA as <30 min/day, 30-60 min/day, and ≥60 min/day (35).

### 2.6 Statistical analysis

Prior to data analysis we scrutinized the data and excluded extreme and erroneous values to guarantee the accuracy of the data analysis. Categorical variables in this study were expressed as percentages. Continuous variables were expressed as mean and standard deviation. Comparisons of categorical variables between adolescents of different genders or with or without the presence of psychological symptoms were performed using the chi-square test. Comparisons of continuous variables across genders were made using t-tests. The associations of SSB consumption and WWI with psychological symptoms in adolescents were analyzed using logistic regression models. Logistic regression analysis was performed with the presence of psychological symptoms in adolescents as the dependent variable and SSB consumption and WWI as the independent variables. Model 1 was the crude model, model 2 adjusted for age, father’s education level, mother’s education level, and family economic level, and model 3 further adjusted for sleep duration, and MVPA. The association between the joint effects of SSB consumption, WWI, and psychological symptoms was analyzed using ordered logistic regression analysis with generalized linear models. The model was adjusted for age, father’s education level, mother’s education level, family economic level, sleep duration, and MVPA. Odds Ratio (OR) and 95% Confidence Interval (CI) were reported separately for the analytic model. Data were analyzed using SPSS 25.0 software for processing and analysis. *P* < 0.05 was used as the test level.

## 3 Results

In this study, 44,675 (22,280 boys, 49.87%) adolescents aged 12-17 years were assessed for SSB consumption, WWI, and psychological symptoms. **Table 1** shows the basic characterization of the Chinese adolescent participants. The results showed that the prevalence of psychological symptoms among Chinese adolescents was 20.9%, and the prevalence of psychological symptoms among boys (21.4%) was higher than that of girls (20.5%), with a statistically significant difference (*χ* ^2^ value of 5.687, *P* < 0.05). The prevalence of emotional problems, behavioral problems, and social adjustment difficulties was 27.7%, 26.7%, and 17.5%, respectively. The percentages of adolescents with SSB consumption <1 times/week, 2-4 times/week, and >4 times/week were 33.4%, 52.1%, and 14.5%, respectively. SSB consumption >4 times/week was higher among boys (17.4%) than girls (11.7%). The WWI of adolescents was 9.36 ± 1.13, and boys 9.42 ± 1.15 had a higher WWI than girls 9.30 ± 1.11, with a statistically significant difference (*t*-value 11.373, *P <*0.001).

**Table 1:** Demographic, anthropometric, and behavioral characteristics of Chinese adolescent participants.

	Boys	Girls	Total	*χ*2/t-value	*P*-value
**Number**	22280	22395	44675		
**Age (years)**	14.64 ± 1.62	14.70 ± 1.65	14.67 ± 1.63	-4.377	<0.001
Weight (Kg, M ± SD)	58.84 ± 13.44	51.20 ± 9.24	55.01 ± 12.14	70.049	<0.001
Waist circumference (cm, M ± SD)	71.69 ± 11.41	66.18 ± 9.17	68.93 ± 10.71	56.258	<0.001
WWI (cm/√kg, M ± SD)	9.42 ± 1.15	9.30 ± 1.11	9.36 ± 1.13	11.373	<0.001
**Father’s education level [N (%)]**				7.050	0.070
Elementary School	2505 (11.2)	2518 (11.2)	5023 (11.2)		
Middle School	7944 (35.7)	7790 (34.8)	15734 (35.2)		
High School	7324 (32.9)	7615 (34.0)	14939 (33.4)		
University and above	4507 (20.2)	4472 (20.0)	8979 (20.1)		
**Mother’s education level [N (%)]**				12.960	0.005
Elementary School	3893 (17.5)	3717 (16.6)	7610 (17.0)		
Middle School	7547 (33.9)	7729 (34.5)	15276 (34.2)		
High School	6788 (30.5)	7038 (31.4)	13826 (30.9)		
University and above	4052 (18.2)	3911 (17.5)	7963 (17.8)		
**Family economic level [N (%)]**				180.064	<0.001
<2000 RMB/month	2367 (10.6)	2571 (11.5)	4938 (11.1)		
2000-5000 RMB/month	7475 (33.6)	8495 (37.9)	15970 (35.7)		
5001-8000 RMB/month	6758 (30.3)	6697 (29.9)	13455 (30.1)		
>8000 RMB/month	5680 (25.5)	4632 (20.7)	10312 (23.1)		
**Sleep duration [N (%)]**				90.396	<0.001
<6 hours/day	3418 (15.3)	3697 (16.5)	7115 (15.9)		
6-8 hours/day	15262 (68.5)	15779 (70.5)	31041 (69.5)		
≥8hours/day	3600 (16.2)	2919 (13.0)	6519 (14.6)		
**MVPA [N (%)]**				1468.437	<0.001
<30 min/day	8465 (38.0)	12052 (53.8)	20517 (45.9)		
30-60 min/day	9620 (43.2)	8303 (37.1)	17923 (40.1)		
≥60 min/day	4195 (18.8)	2040 (9.1)	6235 (14.0)		
**SSB consumption [N (%)]**				499.768	<0.001
<1 times/week	6510 (29.2)	8416 (37.6)	14926 (33.4)		
2-4 times/week	11894 (53.4)	11363 (50.7)	23257 (52.1)		
>4 times/week	3876 (17.4)	2616 (11.7)	6492 (14.5)		
**WWI quartile [N (%)]**				267.431	<0.001
Q1 (<8.82 cm/√kg)	5377 (24.1)	5958 (26.6)	11335 (25.4)		
Q2 (8.82-9.38 cm/√kg)	5238 (23.5)	5971 (26.7)	11209 (25.1)		
Q3 (9.39-9.93 cm/√kg)	5400 (24.2)	5634 (25.2)	11034 (24.7)		
Q4 (>9.93 cm/√kg)	6265 (28.1)	4832 (21.6)	11097 (24.8)		
Emotional problems [N (%)]	6193 (27.8)	6181 (27.6)	12374 (27.7)	0.216	0.643
Behavioral problems [N (%)]	6270 (28.1)	5674 (25.3)	11944 (26.7)	44.889	<0.001
Social adjustment difficulties [N (%)]	4084 (18.3)	3715 (16.6)	7799 (17.5)	23.515	<0.001
Psychological symptoms [N (%)]	4767 (21.4)	4586 (20.5)	9353 (20.9)	5.687	0.017

N, numbers; M, Mean; SD, standard deviation; MVPA, moderate-to-vigorous physical activity; SSB, sugar-sweetened beverage; WWI, weight-adjusted waist index.

**Table 2** shows the comparison of Chinese adolescents with and without the presence of psychological symptoms. The results showed that the adolescents with psychological symptoms had higher weight, waist circumference, and WWI than those without psychological symptoms, and the differences were statistically significant (*t-values* of -9.767, -10.155, and -3.963, *P* < 0.001). The differences in detection rates in terms of SSB consumption and WWI quartile compared to adolescents with or without the presence of psychological symptoms were also both statistically significant (*χ* ^2^ value of 302.625, 28.245, *P* < 0.001).

**Table 2:** Comparison of Chinese adolescents with and without the presence of psychological symptoms.

	Psychological symptoms		*χ* ^2/^ *t*-value	*P*-value
	No	Yes		
**Number**	35322	9353		
**Age (years)**	14.66 ± 1.65	14.69 ± 1.58	-1.667	0.095
Weight (Kg, M ± SD)	54.72 ± 11.95	56.10 ± 12.78	-9.767	<0.001
Waist circumference (cm, M ± SD)	68.66 ± 10.40	69.93 ± 11.75	-10.155	<0.001
WWI (cm/√kg, M ± SD)	9.35 ± 1.11	9.40 ± 1.21	-3.963	<0.001
**Sex [N (%)]**			5.687	0.017
Boys	17513 (78.6)	4767 (21.4)		
Girls	17809 (79.5)	4586 (20.5)		
**Father’s education level [N (%)]**			75.213	<0.001
Elementary School	3776 (75.2)	1247 (24.8)		
Middle School	12366 (78.6)	3368 (21.4)		
High School	11889 (79.6)	3050 (20.4)		
University and above	7291 (81.2)	1688 (18.8)		
**Mother’s education level [N (%)]**			96.912	<0.001
Elementary School	5816 (76.4)	1794 (23.6)		
Middle School	11963 (78.3)	3313 (21.7)		
High School	10968 (79.3)	2858 (20.7)		
University and above	6575 (82.6)	1388 (17.4)		
**Family economic level [N (%)]**			114.200	<0.001
<2000 RMB/month	3622 (73.3)	1316 (26.7)		
2000-5000 RMB/month	12718 (79.6)	3252 (20.4)		
5001-8000 RMB/month	10811 (80.3)	2644 (19.7)		
>8000 RMB/month	8171 (79.2)	2141 (20.8)		
**Sleep duration [N (%)]**			702.744	<0.001
<6 hours/day	4827 (67.8)	2288 (32.2)		
6-8 hours/day	24973 (80.5)	6068 (19.5)		
≥8hours/day	5522 (84.7)	997 (15.3)		
**MVPA [N (%)]**			255.242	<0.001
<30 min/day	15540 (75.7)	4977 (24.3)		
30-60 min/day	14635 (81.7)	3288 (18.3)		
≥60 min/day	5147 (82.6)	1088 (17.4)		
**SSB consumption [N (%)]**			302.625	<0.001
<1 times/week	12092 (81.0)	2834 (19.0)		
2-4 times/week	18620 (80.1)	4637 (19.9)		
>4 times/week	4610 (71.0)	1882 (29.0)		
**WWI quartile [N (%)]**			28.245	<0.001
Q1	9000 (79.4)	2335 (20.6)		
Q2	8985 (80.2)	2224 (19.8)		
Q3	8750 (79.3)	2284 (20.7)		
Q4	8587 (77.4)	2510 (22.6)		

N, numbers; M, Mean; SD, standard deviation; MVPA, moderate-to-vigorous physical activity; SSB, sugar-sweetened beverage; WWI, weight-adjusted waist index.

**Table 3** shows a one-way comparison of SSB consumption, WWI, and psychological symptoms among Chinese adolescents. The results showed that overall, the differences in the prevalence of emotional problems, behavioral problems, social adjustment difficulties, and psychological symptoms among Chinese adolescents with different SSB consumption were all statistically significant (*χ* ^2^ values of 278.418, 287.364, 297.538, 302.625, *P* < 0.001). The prevalence of emotional problems, behavioral problems, social adjustment difficulties, and psychological symptoms among WWI quartile adolescents was also statistically significant (*χ* ^2^ values of 24.900, 28.133, 12.522, 28.245, *P* < 0.05).

**Table 3:** A one-way comparison of SSB consumption, WWI, and psychological symptoms among Chinese adolescents.

	N	Emotional problems			Behavioral problems			Social adjustment difficulties			Psychological symptoms		
		N (%)	χ^2^-value	*P-*value	N (%)	χ^2^-value	*P*-value	N (%)	χ^2^-value	*P*-value	N (%)	χ^2^-value	*P*-value
Boys													
**SSB consumption [N(%)]**			122.711	<0.001		103.867	<0.001		128.114	<0.001		125.340	<0.001
<1 times/week	6510	1722(26.5)			1732(26.6)			1135(17.4)			1293(19.9)		
2-4 times/week	11894	3113(26.2)			3188(26.8)			1992(16.7)			2385(20.1)		
>4 times/week	3876	1358(35.0)			1350(34.8)			957(24.7)			1089(28.1)		
**WWI quartile [N(%)]**			7.026	0.071		7.641	0.054		10.981	0.012		14.016	0.003
Q1	5377	1535(28.5)			1552(28.9)			1065(19.8)			1108(20.6)		
Q2	5238	1399(26.7)			1401(26.7)			923(17.6)			1057(20.2)		
Q3	5400	1472(27.3)			1515(28.1)			983(18.2)			1176(21.8)		
Q4	6265	1787(28.5)			1802(28.8)			1113(17.8)			1426(22.8)		
Girls													
**SSB consumption [N(%)]**			167.350	<0.001		180.344	<0.001		166.727	<0.001		182.718	<0.001
<1 times/week	8416	2118(25.2)			1942(23.1)			1270(15.1)			1541(18.3)		
2-4 times/week	11363	3070(27.0)			2793(24.6)			1781(15.7)			2252(19.8)		
>4 times/week	2616	993(38.0)			939(35.9)			664(25.4)			793(30.3)		
**WWI quartile [N(%)]**			21.631	<0.001		21.338	<0.001		7.562	0.056		16.874	0.001
Q1	5958	1675(28.1)			1518(25.5)			1020(17.1)			1227(20.6)		
Q2	5971	1578(26.4)			1424(23.8)			954(16.0)			1167(19.5)		
Q3	5634	1485(26.4)			1397(24.8)			896(15.9)			1108(19.7)		
Q4	4832	1443(29.9)			1335(27.6)			845(17.5)			1084(22.4)		
**Total**													
**SSB consumption [N(%)]**			278.418	<0.001		287.364	<0.001		297.538	<0.001		302.625	<0.001
<1 times/week	14926	3840(25.7)			3674(24.6)			2405(16.1)			2834(19.0)		
2-4 times/week	23257	6183(26.6)			5981(25.7)			3773(16.2)			4637(19.9)		
>4 times/week	6492	2351(36.2)			2289(35.3)			1621(25.0)			1882(29.0)		
**WWI quartile [N(%)]**			24.900	<0.001		28.133	<0.001		12.522	0.006		28.245	<0.001
Q1	11335	3210(28.3)			3070(27.1)			2085(18.4)			2335(20.6)		
Q2	11209	2977(26.6)			2825(25.2)			1877(16.7)			2224(19.8)		
Q3	11034	2957(26.8)			2912(26.4)			1879(17.0)			2284(20.7)		
Q4	11097	3230(29.1)			3137(28.3)			1958(17.6)			2510(22.6)		

N, numbers; SSB, sugar-sweetened beverage; WWI, weight-adjusted waist index.

**Table 4** shows the multivariate logistic regression analyses of SSB consumption, WWI, and psychological symptoms among Chinese adolescents. Multiple logistic regression analyses were conducted with the presence of psychological symptoms as the dependent variable and SSB consumption and WWI as the independent variables, respectively, stratified by sex. Overall, the results showed that adolescents with SSB consumption >4 times/week had 1.62 times (95% CI: 1.50-1.74) the risk of developing psychological symptoms than those in the group with SSB consumption <1 times/week (*P* < 0.001). Adolescents in the WWI quartile of Q4 had 1.15 times (95% CI: 1.07-1.22) the risk of developing psychological symptoms than adolescents in the Q1 group (*P* < 0.001). The same trend was observed in boys and girls.

**Table 4:** Multivariate logistic regression analysis of SSB consumption, WWI, and psychological symptoms in Chinese adolescents.

Sex/Variable	Group	Psychological symptoms					
		Model 1		Model 2		Model 3	
		OR (95% CI)	*P-*value	OR (95% CI)	*P-*value	OR (95% CI)	*P-*value
Boys							
**SSB consumption [N (%)]**	<1 times/week	1.00		1.00		1.00	
	2-4 times/week	1.01 (0.94~1.09)	0.733	1.02 (0.94~1.10)	0.658	1.01 (0.93~1.09)	0.860
	>4 times/week	1.58 (1.44~1.74)	<0.001	1.62 (1.48~1.78)	<0.001	1.46 (1.32~1.61)	<0.001
**WWI quartile [N (%)]**	Q1	1.00		1.00		1.00	
	Q2	0.97 (0.89~1.07)	0.585	0.98 (0.89~1.07)	0.635	0.99 (0.90~1.09)	0.779
	Q3	1.07 (0.98~1.18)	0.137	1.08 (0.99~1.19)	0.103	1.10 (1.00~1.20)	0.055
	Q4	1.14 (1.04~1.24)	0.005	1.16 (1.06~1.26)	0.002	1.17 (1.07~1.28)	0.001
Girls							
**SSB consumption [N (%)]**	<1 times/week	1.00		1.00		1.00	
	2-4 times/week	1.10 (1.03~1.19)	0.007	1.10 (1.02~1.18)	0.010	1.04 (0.97~1.13)	0.252
	>4 times/week	1.95 (1.77~2.16)	<0.001	2.00 (1.81~2.21)	<0.001	1.73 (1.56~1.93)	<0.001
**WWI quartile [N (%)]**	Q1	1.00		1.00		1.00	
	Q2	0.94 (0.86~1.02)	0.152	0.94 (0.86~1.03)	0.167	0.95 (0.87~1.04)	0.237
	Q3	0.94 (0.86~1.03)	0.213	0.94 (0.86~1.03)	0.186	0.95 (0.87~1.05)	0.315
	Q4	1.12 (1.02~1.22)	0.020	1.10 (1.01~1.21)	0.040	1.11 (1.01~1.22)	0.034
Total							
**SSB consumption [N (%)]**	<1 times/week	1.00		1.00		1.00	
	2-4 times/week	1.06 (1.01~1.12)	0.022	1.06 (1.01~1.12)	0.021	1.04 (0.99~1.10)	0.134
	>4 times/week	1.75 (1.63~1.87)	<0.001	1.79 (1.67~1.92)	<0.001	1.62 (1.50~1.74)	<0.001
**WWI quartile [N (%)]**	Q1	1.00		1.00		1.00	
	Q2	0.95 (0.89~1.02)	0.156	0.96 (0.90~1.02)	0.182	0.96 (0.90~1.03)	0.273
	Q3	1.01 (0.94~1.07)	0.854	1.01 (0.94~1.08)	0.828	1.02 (0.96~1.09)	0.513
	Q4	1.13 (1.06~1.2)	<0.001	1.13 (1.06~1.20)	<0.001	1.15 (1.07~1.22)	<0.001

N, numbers; SSB, sugar-sweetened beverage; WWI, weight-adjusted waist index. OR, Odds Ratio; 95% CI, 95% Confidence Interval.

**Figure 2:**
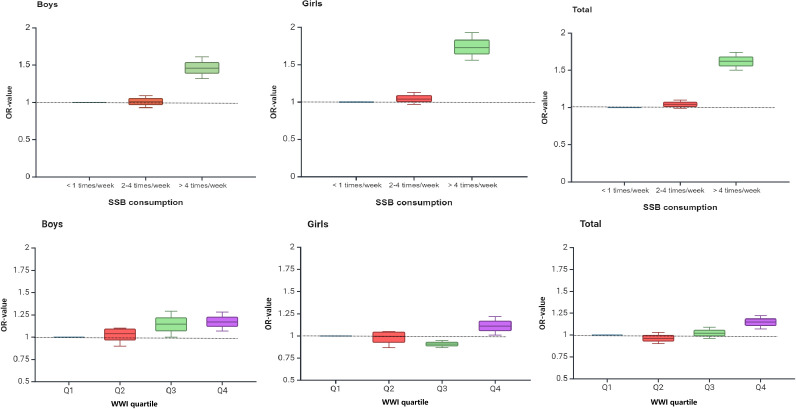
Trends in ORs of multiple logistic regression analysis of SSB consumption, WWI, and psychological symptoms among Chinese adolescents.

**Figure 2** shows the trend of OR values of SSB consumption, WWI quartile, and psychological symptoms in Chinese adolescents by multivariate logistic regression analysis. As can be seen from the figure, with the increase in SSB consumption and WWI quartile, the overall trend of OR value was higher.

**Table 5** shows the ordered logistic regression analysis of SSB consumption, WWI quartile, and psychological symptoms among Chinese adolescents. The presence of psychological symptoms among adolescents was used as the dependent variable, and different combinations of SSB consumption and WWI quartile were used as independent variables. Overall, the results showed that adolescents in the group with SSB consumption <1 times/week and WWI quartile Q1 as the reference group, and adolescents in the group with SSB consumption >4 times/week and WWI quartile Q4 had the highest risk of developing psychological symptoms (OR=2.01, 95% CI:1.76-2.30) (*P <*0.001). The same trend was observed for boys (OR=1.67, 95% CI:1.39-2.00) and girls (OR=2.68, 95% CI:2.17-3.31) (*P <*0.001).

**Table 5:** Ordered logistic regression analysis of SSB consumption, WWI, and psychological symptoms among Chinese adolescents.

Sex	Classification of interaction		Psychological symptoms	
	SSB consumption [N (%)]	WWI quartile [N (%)]	OR (95% CI)	*P*-value
**Boys**	<1 times/week	Q1	1.00	
		Q2	0.89 (0.74~1.07)	0.224
		Q3	1.06 (0.89~1.27)	0.482
		Q4	1.06 (0.89~1.25)	0.521
	2-4 times/week	Q1	0.96 (0.82~1.12)	0.628
		Q2	0.91 (0.78~1.07)	0.254
		Q3	1.04 (0.89~1.22)	0.589
		Q4	1.14 (0.98~1.33)	0.082
	>4 times/week	Q1	1.45 (1.20~1.75)	<0.001
		Q2	1.68 (1.39~2.03)	<0.001
		Q3	1.55 (1.28~1.88)	<0.001
		Q4	1.67 (1.39~2.00)	<0.001
**Girls**	<1 times/week	Q1	1.00	
		Q2	0.96 (0.82~1.12)	0.628
		Q3	0.96 (0.82~1.12)	0.565
		Q4	1.20 (1.03~1.41)	0.022
	2-4 times/week	Q1	1.13 (0.98~1.31)	0.085
		Q2	1.09 (0.95~1.26)	0.224
		Q3	1.11 (0.96~1.28)	0.172
		Q4	1.18 (1.02~1.37)	0.027
	>4 times/week	Q1	1.92 (1.60~2.31)	<0.001
		Q2	1.73 (1.41~2.12)	<0.001
		Q3	1.82 (1.48~2.24)	<0.001
		Q4	2.68 (2.17~3.31)	<0.001
**Total**	<1 times/week	Q1	1.00	
		Q2	0.93 (0.83~1.05)	0.237
		Q3	1.00 (0.89~1.13)	0.959
		Q4	1.14 (1.01~1.27)	0.028
	2-4 times/week	Q1	1.05 (0.95~1.17)	0.330
		Q2	1.01 (0.91~1.12)	0.878
		Q3	1.08 (0.98~1.20)	0.134
		Q4	1.18 (1.06~1.31)	0.002
	>4 times/week	Q1	1.67 (1.47~1.91)	<0.001
		Q2	1.73 (1.51~1.99)	<0.001
		Q3	1.69 (1.47~1.94)	<0.001
		Q4	2.01 (1.76~2.30)	<0.001

N, numbers; SSB, sugar-sweetened beverage; WWI, weight-adjusted waist index. OR, Odds Ratio; 95% CI, 95% Confidence Interval.

## 4 Discussion

To the best of our knowledge, this study is the first to analyze the association between SSB consumption, and WWI with psychological symptoms in Chinese adolescents using a national sample. The present study showed that the prevalence of psychological symptoms among Chinese adolescents was 20.9%, which was higher than the results of related studies (17.9%), but also lower than the results of some studies (21.4%) (36, 37). The reasons for this exist in several ways: Firstly, there are some differences in the assessment questionnaires used in different studies, leading to inconsistent results between studies. Second, there are also some differences in the choice of region and age distribution of participants in different studies, which are also important reasons for the differences in results. Thirdly, there are differences in the time of investigation in different studies, which also contributes to the differences in adolescent psychological symptoms. There is an inconsistency between the present study and related studies in that the prevalence of psychological symptoms in Chinese adolescent boys was higher than that of girls in the present study. Many past studies have shown that adolescent girls have a higher prevalence of psychological symptoms compared to boys, and the findings of the present study are inconsistent with these findings (38, 39). First, we believe that boys are often expected to be “strong” and “rational”, and that boys may be more inclined to hide their emotional problems, which may result in psychological problems not being resolved promptly, leading to a higher prevalence of psychological symptoms in boys than in girls (40). This may lead to psychological problems not being solved in time, resulting in a higher prevalence of psychological problems in boys than in girls. Secondly, social role expectations lead to harsher requirements for boys (41). Society generally believes that girls should be more gentle and rational, and this invisible gender role expectation negatively affects boys’ self-identity, which in turn leads to the emergence of psychological symptoms (41). Finally, this study also shows that boys have higher WWI values than girls, and higher WWI indicates an association with obesity, leading to a higher prevalence of psychological symptoms in boys than in girls (42).

The results of the present study also showed that adolescents with higher SSB consumption were at higher risk of developing psychological symptoms, and the results of the present study are consistent with the findings of several past studies. First, elevated SSB consumption is an important risk factor for obesity, and there is a strong association between the development of obesity and the development of psychological symptoms (43). Secondly, the increase in SSB consumption leads to changes in the intestinal flora of adolescents, causing hormonal disorders in the body and a decrease in the secretion of dopamine, which can lead to the occurrence of bad moods, thus leading to the emergence of various types of psychological problems (44). Third, SSB consumption induces glucose metabolism disorders and insulin resistance, and such metabolic disorders cause mild but persistent inflammatory responses in the body, and the release of some inflammatory factors itself increases the risk of depression, leading to the development of psychological symptoms (45). In addition, it has been found that increased SSB consumption leads to a decrease in nerve growth factor and serotonin receptors, which leads to disturbances in the feedback control of serotonin synthesis and release in the hypothalamus, and a decrease in the ability to cope with stress, which can also lead to psychological problems (46).

The results of the present study also show that there is an association between increased WWI and increased prevalence of psychological symptoms in Chinese adolescents. The study showed a significant positive correlation between increased WWI and increased suicidal ideation, characterized by a nonlinear relationship that remained in the adjusted models (47). The underlying causes are related to the hormonal secretion and inflammatory response of the body due to obesity. It has been found that the accumulation of visceral fat, especially abdominal fat, leads to insulin resistance, which in turn leads to an increase in chronic diseases and inflammatory factors, which can lead to psychological stress or emotional problems (48). It has also been shown that increased WWI contributes to the emergence of obesity, and obese individuals are often accompanied by higher levels of inflammation, which may affect mental health through disturbance in neuroimmune regulatory mechanisms (49). In addition, it has been found that inflammatory factors may directly affect brain function, leading to impaired emotion regulation, which increases the risk of psychological symptoms (50).

Overall, the ordered logistic regression analysis of this study showed that adolescents with SSB consumption >4 times/week and WWI quartile of Q4 had the highest risk of developing psychological symptoms, and the same trend was observed in boys and girls. This suggests that there is a joint effect of SSB consumption and WWI on psychological symptoms. An increase in SSB consumption further leads to an increase in body weight, which increases the risk of obesity, and the occurrence of obesity increases the value of WWI, which leads to an increase in the prevalence of psychological symptoms (51). We believe that the increased prevalence of psychological symptoms due to increased SSB consumption and elevated WWI values is associated with social factors in addition to intrinsic hormonal disorders and inflammatory responses. Studies have shown that excessive waist circumference in adolescents may result in low self-esteem and anxiety due to external body image problems, and may even trigger depressive symptoms (52). Societal prejudice against body size may result in individuals with excessively large waist circumference facing more pressure and discrimination in social situations, all of which may hurt adolescents’ mental health and cause psychological symptoms (53). In addition, increased waist circumference may be associated with an unhealthy diet and lack of exercise, and an unhealthy lifestyle may cause individuals to feel guilty and powerless, these negative emotions can affect mental health and cause psychological symptoms (54).

There are certain strengths and limitations of this study. Strengths: First, to the best of our knowledge, this study analyzed the association between SSB consumption, WWI, and psychological symptoms for the first time using a national sample of Chinese adolescents, which provides a reference and help for the intervention and prevention of adolescent psychological symptoms. Second, the sample size of this study is relatively large and the findings are representative. However, this study also has some limitations. First, this study is a cross-sectional investigation, which can only analyze the association relationship between SSB consumption, WWI, and the existence of psychological symptoms, but not the causal relationship. Second, the covariates included in this study were limited, and risk factors such as smoking, alcoholism and academic stress that affect adolescent psychological symptoms should be included in the future to analyze the results more accurately. Third, this study adopts the method of reminiscence questionnaire to assess the participants’ SSB consumption, which is affected by the participants’ recall ability, and inevitably has some deviation from the reality, which is also one of the limitations of this study. Fourth, the total caloric intake or other dietary factors of the participants were not considered in this study, which may also lead to certain biases in the analysis results and is also a limitation of this study. In addition, this study only investigated adolescents in the age group of 12-17 years old and did not involve students in elementary school; more age groups should be included for future investigation and analysis.

## 5 Conclusions

Positive associations were found between SSB consumption, WWI, and psychological symptoms in Chinese adolescents. Increased SSB consumption and WWI both lead to increased prevalence of psychological symptoms in adolescents. In the future, the control of SSB consumption and WWI should be taken into account in the process of adolescent psychosocial symptoms education and intervention to better reduce the prevalence of adolescent psychosocial symptoms.

## Data Availability

The raw data supporting the conclusions of this article will be made available by the authors, without undue reservation.
